# Transient Effects of Snow Cover Duration on Primary Growth and Leaf Traits in a Tundra Shrub

**DOI:** 10.3389/fpls.2022.822901

**Published:** 2022-04-05

**Authors:** Lucrezia Unterholzner, Angela Luisa Prendin, Raffaella Dibona, Roberto Menardi, Valentino Casolo, Sara Gargiulo, Francesco Boscutti, Marco Carrer

**Affiliations:** ^1^Department of Land Environment Agriculture and Forestry, University of Padova, Legnaro, Italy; ^2^Department of Biology, Ecoinformatics and Biodiversity, Aarhus University, Aarhus, Denmark; ^3^Department of Agricultural Food Environmental Animal Sciences, University of Udine, Udine, Italy; ^4^Department of Life Sciences, University of Trieste, Trieste, Italy

**Keywords:** snowmelt, alpine tundra, shrub phenology, *Juniperus communis*, leaf traits, non-structural carbohydrates, primary growth, climate change

## Abstract

With the recent climate warming, tundra ecotones are facing a progressive acceleration of spring snowpack melting and extension of the growing season, with evident consequences to vegetation. Along with summer temperature, winter precipitation has been recently recognised as a crucial factor for tundra shrub growth and physiology. However, gaps of knowledge still exist on long-living plant responses to different snowpack duration, especially on how intra-specific and year-to-year variability together with multiple functional trait adjustments could influence the long-term responses. To fill this gap, we conducted a 3 years snow manipulation experiment above the Alpine treeline on the typical tundra species *Juniperus communis*, the conifer with the widest distributional range in the north emisphere. We tested shoot elongation, leaf area, stomatal density, leaf dry weight and leaf non-structural carbohydrate content of plants subjected to anticipated, natural and postponed snowpack duration. Anticipated snowpack melting enhanced new shoot elongation and increased stomatal density. However, plants under prolonged snow cover seemed to compensate for the shorter growing period, likely increasing carbon allocation to growth. In fact, these latter showed larger needles and low starch content at the beginning of the growing season. Variability between treatments slightly decreased over time, suggesting a progressive acclimation of juniper to new conditions. In the context of future warming scenarios, our results support the hypothesis of shrub biomass increase within the tundra biome. Yet, the picture is still far from being complete and further research should focus on transient and fading effects of changing conditions in the long term.

## Introduction

Arctic and Alpine regions are currently experiencing higher warming rates than the global average and this trend is likely to increase in the near future, especially during winter periods (Hoegh-Guldberg et al., 2018). Temperature variations, together with decreasing frequencies of cold extremes and local alteration of the precipitation pattern (Allan et al., 2021), deeply affect the cryosphere. For example, with each decade from the 1980s, the Arctic has shown a reduction in spring (May) snow cover extent of ca. 10% and a reduction in April snow mass of 5% (Richter-Menge et al., 2016). This also induced an advance of the snow-free period by ca. 5 days per decade in the Northern Hemisphere (Dye, 2002; Bormann et al., 2018). As a result, there are already significant, cascading effects on plant communities. Species composition, distribution and relative abundance, and species traits, such as plant height, biomass allocation and canopy cover, have been altered (Myers-Smith et al., 2011), with a significant effect on plant diversity (Boscutti et al., 2018). These changes result in heterogeneous rates of Alpine and Arctic tundra greenness (Berner et al., 2020; Myers-Smith et al., 2020) and phenological responses, and in an overall shrubline advance around the globe observed in the last decades (Dial et al., 2015; Carlson et al., 2017; Myers-Smith and Hik, 2018; Steinbauer et al., 2018; Frost et al., 2020). Among the tundra systems, the European Alps at high elevation may be even more vulnerable than the Arctic, due to differences in the photoperiod and resource availability (Ernakovich et al., 2014). In parallel with northern hemisphere tendency, by the end of the twenty-first century, above the alpine treeline is predicted to see a reduction of 30–80% of snow water equivalent and an acceleration of spring snowpack melting by up to 50–60 days (Beniston et al., 2003; Steger et al., 2013). Nevertheless, the key mechanisms driving the response of plant communities to these shifting scenarios are still poorly understood, leaving uncertainties on the possible future tundra-vegetation feedback. This calls for the importance of deeply understanding the sensitivity of morphological and physiological responses of tundra species under varying climate conditions. This is especially true for shrubs species, the dominant long-lived organisms above the treeline, of which survival and fitness depend more on their capacity to acclimate/adapt to changing conditions, rather than to migrate to more fitting sites. Plants with slow regeneration times, e.g., *Juniperus communis* (*J. communis*) (García et al., 1999; Gruwez et al., 2017; Jacquemart et al., 2021) or scarce dispersal capacity might be forced to face the environmental changes, being hindered in migration (Midgley et al., 2006; Aitken et al., 2008; Sedlacek et al., 2015).

Tundra represents a typical heat-limited environment, where most of the investigations on climate change effects on vegetation focus on rising temperature, overlooking the role of changing snow cover dynamics (Dormann and Woodin, 2002; Walker et al., 2006; Elmendorf et al., 2012; Bjorkman et al., 2018). However, the date of snow melting is deemed a key factor driving the seasonal radial growth of trees (Kirdyanov et al., 2003). Recent studies demonstrated that, besides summer-temperature, winter precipitation can play a crucial role in shrub growth, opening new questions on the association between snow and shrub dynamics (Hallinger et al., 2010; Pellizzari et al., 2014; Martin et al., 2017; Carrer et al., 2019; Pandey et al., 2020). In fact, in heat limited environments, close-to-soil shrubs, with aboveground meristems below the boundary layer, may benefit from warmer microclimate conditions in respect to trees that have air-exposed meristems (Körner, 2012). Still, the influence of winter-precipitation on shrub performance is controversial due to the interaction of several effects: snow cover can provide protection against frost and soil nutrient losses, and ensure water availability but, at the same time, when abundant, can shorten the growing season, hindering growth or altering phenological dynamics (Hallinger et al., 2010; Rixen et al., 2010; Francon et al., 2017; Carrer et al., 2019; Gao et al., 2021).

So far, investigations targeting snow cover effects mainly explore single plant’s parameters as radial annual growth or shoot elongation (Rixen et al., 2010; Schmidt et al., 2010; Pellizzari et al., 2014; Francon et al., 2017; Carrer et al., 2019), while other key functional traits or physiological parameters crucial for plant fitness, productivity, and survival, as leaf traits or non-structural carbohydrate content, are mostly neglected, especially in shrub-related studies (Wipf et al., 2009; Sedlacek et al., 2015; Filippi et al., 2021). In fact, leaves and stomata features, driving photosynthesis and transpiration processes, reflect plant functioning in specific growing conditions (Chapin et al., 2002). Similarly, non-structural carbohydrates (NSCs) content, that is the pool of photosynthetic products suitable for growth, metabolism maintenance and defence, are pivotal to understanding plant response to environmental drivers (Kozlowski, 1992; Martínez-Vilalta et al., 2016). In particular, NSCs have been intensively studied at the treeline with regard to the growth limitation hypothesis (Hoch et al., 2002; Fajardo et al., 2012; Hoch and Körner, 2012; Fajardo and Piper, 2014). Recently, these investigations, on the role of NSC in response to elevational gradient, have been extended to shrubs (Dolezal et al., 2019) and dwarf shrubs (Casolo et al., 2020). However, only a few studies, with contrasting results, compared NSC content with snow depth and cover length (Palacio et al., 2015; Wheeler et al., 2016; Domisch et al., 2017).

Among the long-living shrubs, the common juniper (*J. communis* L.) is a cold and drought tolerant pioneer species with the widest distributional range across the northern hemisphere, representing one of the most typical tundra woody species at high latitude and elevation (Adams, 2014; Enescu et al., 2016). Hence, it represents a model species to investigate physiological responses of woody species to environmental variability above the treeline ecotone. To assess the responses of *J. communis* to snow cover duration, we established a snow manipulation experiment above the treeline in the Italian Alps. Primary growth, leaf traits and leaf NSC content were measured for three years to test the effects of different snowmelt timings on common juniper axial growth and carbon gain. Specifically, we expected: (i) a short-term and cumulative (across years) increasing effect of snow cover duration on shrub primary growth with the positive effect of early snowmelt and negative for late snowmelt as a response to longer/shorter growing season; (ii) a higher risk of early frost events in photosynthetic organs with earlier snowmelt, reflected by a higher concentration of soluble NSC in leaves and (iii) a significant influence on leaf traits (e.g., leaf size, dry weight and stomatal density) as the new needles tend to rapidly adjust to maximise light exposure and gas exchange for the different vegetation period.

## Materials and Methods

### Study Site and Experimental Design

**FIGURE 1:**
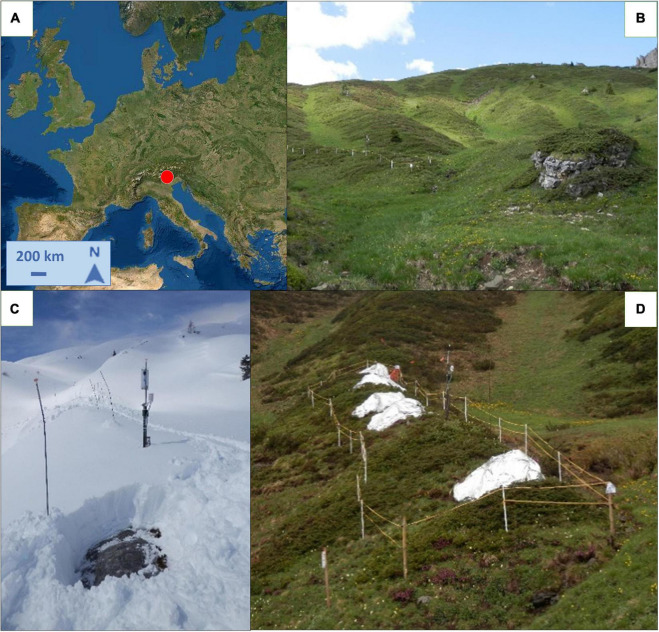
**(A)** Location (46°29′9′′N; 12° 3′21′′E, 2,155 m a.s.l.) and **(B)** Overview of the study site. **(C)** Early snow melting treatment where snowpack had been removed and juniper was early air-exposed. **(D)** Late snow melting treatment with snowpack protected by thermal sheets. Sources: **(A)** Esri World Topo Map.

The area is located above the treeline nearby Giau pass in the Dolomites, Italy (46°29′9′′N; 12° 3′21′′E, 2155 m a.s.l.; Figure 1A). The substrate is siliceous and soils are deep leptosol (ARPAV-Servizio Osservatorio Suolo e Bonifiche, 2011). We selected an east-facing gentle slope (ca. 20°) with morphological homogeneity and abundance of *J. communis* individuals growing on early snow-free mounds (Figure 1B). Within the area, we defined a plot of 30 m × 4 m, almost fully covered by the target species, and identified 15 shrubs clearly distinguished from each other. We delimited the area with a fence to avoid livestock grazing and trampling damage. We applied three different treatments, each on five randomly distributed individuals (experimental blocks) within the plot: the early snow melting, (E), from which we removed snow before the natural melting period (Figure 1C); the control (C), where snowpack remained untouched and snow melted naturally according to yearly precipitation and temperature regimes; and last, the late snow melting (L), on which we prolonged the snow cover as much as possible (Figure 1D).

The day of snowpack removal on the E-plants was modulated year by year according to the meteorological conditions and snow abundance that was monitored through a webcam located within the study site and regular visits to the site. In contrast, to prolong the snow cover on the L-plants, we manually accumulated snow and applied reflective insulating thermal sheets (padding in aluminium and polyester) ca. 1.5 mm thick, to postpone the melting (Figure 1C).

The experiment was setup in early autumn 2017 and conducted continuously during the three following years 2018, 2019, and 2020.

### Time Series of Climate and Microsite Condition

A meteorological station was set up within the study site to record air temperature at 2 m above the ground, through Type T Thermocouple (error ± 0.5°C). The volumetric soil water content was also measured every hour through Time Domain Reflectometry (TDR) method, with probes that were previously calibrated for the soil type (precision ± 2%; Campbell Scientific co. Mo. SC615). Snow depth records were automatically collected every 30 min from 1988 at the close Falzarego Pass (46°31′10′′N; 12° 0′33′′E, 2090 m a.s.l.) and manually every 1 or 2 weeks at Giau Pass station (46°29′9′′N; 12° 3′21′′E, 2155 m a.s.l.; ARPA Veneto—Centro Valanghe di Arabba). Since data from the two stations were significantly correlated (*r* = 0.99), and Falzarego Pass snow-depth data have higher resolution and longer historical record, we used the Falzarego Pass data.

### Primary Growth Measurement

New shoot elongation of common juniper individuals was measured with a calliper every 7–10 days from the moment they sprouted from the buds (between May and June according to snow cover duration) until they reached the maximum length (September). Branches and shoots were randomly selected and labelled with coloured tags to be identified from one season to the next. Sample number increased over years from 1 branch × 1 shoot × individual (*n* = 15 shoots) in 2018 to 3 branch × 3 shoots × individual (*n* = 135) and finally to 6 branches × 3 shoots (*n* = 270) in 2020.

### Leaf Traits

At the end of September 2020, we collected from each individual ca. 30 to 50 mature and healthy needles from the last 3 yearly internodes. We used 225 needles for the stomatal density analysis (5 needles × 3 years × 15 shrubs): we imprinted the lower side of the needles with nail varnish, then we peeled off the rubber with an adhesive tape, placed it on a glass slide and collected digital images of the stomatal imprints at 10X with a NIKON Eclipse 80i microscope. Then with an image analysis software (GIMP 2.10), we counted the number of stomata for a fixed 1 mm^2^ area.

The remaining needles were then used to define leaf area, using WinSEEDLE (V 5.0) image analysis system, and the dry weight. Dry weight was assessed on needles left 24 h in the oven at 50°C.

Finally, specific leaf area (SLA) was computed as the ratio between needle area and dry weight.

### Non-structural Carbohydrates

To explore the variation of NSC content, we collected samples of leaves from 2- to 3-year-old branches of 15 plants at the beginning (June) and end (September) of each growing season. Sampling occurred between 11.00 and 12.00 a.m. on sunny days to avoid diurnal variability and plant material was immediately stored in a plastic bag inside a cool portable fridge, brought to the lab and microwaved for 3 min at 700 W to stop carbohydrate consumption. Then, we ground leaves (particles size less to 0.15 mm) with liquid nitrogen and dried them in an oven at 55°C overnight. Soluble NSC and starch content were extracted following the method proposed by Quentin et al. (2015) and Landhäusser et al. (2018). About 15 mg of ground samples were weighed into Eppendorf tubes (2 ml volume) and incubated in 0.5 ml of 80% (v/v) ethanol for 30 min at 80°C. Samples were centrifuged at 14,000 rpm for 3 min (with Mikro 120, Hettuch zentrifugen) and supernatants were transferred into new Eppendorf tubes (1.5 ml volume). A second incubation of pellets was performed with 0.5 ml ethanol. Then we added the resulting supernatant to the first and dried it in the oven until ethanol evaporation was complete. Crystallised soluble NSCs were suspended in 0.5 ml of 50 mM of Tris-HCl pH 7.5 and stored at −20°C until the application of anthrone assay (Yemm and Willis, 1954). This method allows for quantifying NSC content by means of spectrophotomertic measurements by converting absorbance at 620 nm, in the amount of glucose (g/g DW) through a titration curve obtained with known amounts of glucose.

For starch quantification, the pellet coming from sugar extraction was suspended in 1 ml of 0.2 M sodium acetate trihydrate (pH 4.6) and subjected to gelatinisation by boiling it for 1 h. Starch enzymatic hydrolysis to glucose was induced by means of 100 U of α-amylase and 25 U of amyloglucosidase per sample. The reaction was performed at 55°C in an oven overnight, then samples were boiled for five minutes to interrupt the enzymatic activity. Glucose derived from starch hydrolysis was evaluated as NADPH formation following the method proposed by Bergmeyer and Bernt (1974) by means of 0.3 U of Hexokinase and 0.5 U of Glucose-6-phosphate dehydrogenase, in buffer solution (50 mM Tris-HCl, 2 M MgCl_2_, 50 mM NADP^+^ and 0.4 M NaATP) at 32°C. NADPH produced was read at spectrophotometer at 340 nm and converted into glucose through a titration curve.

All the spectrophotometric measurements were performed with VICTOR3 Multilabel Counter Plate Reader (Perkin Elmer, Boston, MA, United States).

### Statistical Analysis

Data of all variables were tested for normality and homoscedasticity, through the Shapiro–Wilk and Levene tests. To detect air temperature, soil water content and snow depth trends, we averaged data by the day of the year (doy) for 2018, 2019, and 2020 and interpolated them through a spline function.

In the case of non-normal distribution, variables were square-root transformed, except for shoot elongation for which requirements were not satisfied even after transformation. For all variables, except for new shoot elongation, differences between treatments for single years and for the average of the whole period were tested through linear mixed-effects models (nested ANOVA), accounting for the treatment as a fixed factor and experimental block as a random factor. We used the nlme R package (Pinheiro et al., 2021). Then, we run a pairwise comparison on the same models *via* the emmeans package in R (Lenth, 2022). Finally, to analyse shoot elongation, a generalised additive model (GAM) was used, through the mgcv R package (Wood, 2017), according to the following formula:


shoot_elongation=s(doy,by=treatment)+year+treatment+(individual/stem/branch)

Where shoot elongation is modelled according to the interaction between the day of the year (doy) and treatment with year and specimen as covariates. Specimens (individual, stem and branch) are treated as nested parameters being one subgroup of the other; s represents the smoothing function that can be linear or non-linear (Wood, 2017). Also, for shoot elongation, both single years and the average for the period 2018–2020 were considered. The maximum elongation rate and the beginning time of elongation were derived from the GAM model, considering all single measurements together. All statistical analyses were performed using R software (R Core Team, 2021).

## Results

### Climate Data and Growing Season Length

Daily mean air temperature recorded at the experimental site followed the typical seasonal trend, peaking between mid-July and August with a value of 12.2°C and a minimum of −6.7°C in January. Daily soil water content was rather constant until mid-March and later showed two peaks, in mid-May (33.05%) and at the beginning of November (30.22%; Supplementary Figure 1A).

In the three years of the experiment, the snow melted 10–35 days earlier than usual respective to the average (1988–2021) melting date occurring at the beginning of June. Yet, snowpack depth was thicker than usual in 2018 and 2020, with 122 and 112 cm, respectively, while in 2019 it was 70 cm, equal to the average value of the whole monitoring 1988–2021 period (Supplementary Figure 1B).

**FIGURE 2:**
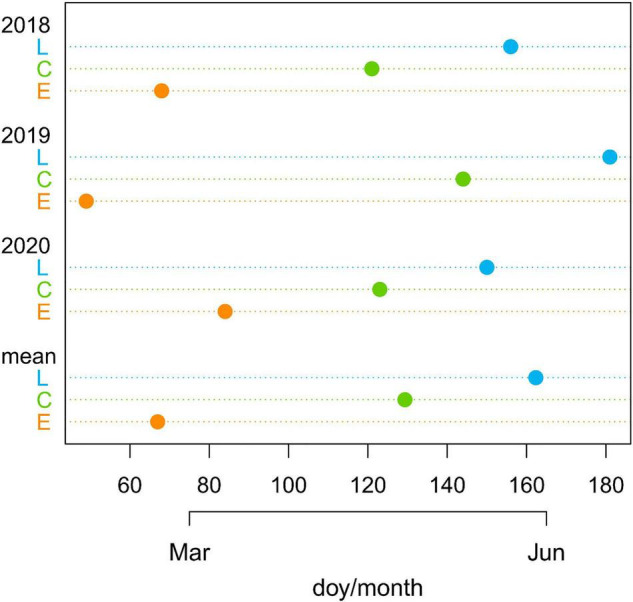
Day of the year (doy) with indication of months when common junipers for the three treatments (late-melt L, control C, and early-melt E) emerged from the snow cover in 2018, 2019, and 2020 together with the mean values for the 3 years.

Considering the average value of the three years, E-plants were air-exposed 62 days before the controls, while L plants were air-exposed 33 days after the controls (Figure 2 and Supplementary Table 1).

### Primary Growth Trend During the Growing Season

**FIGURE 3:**
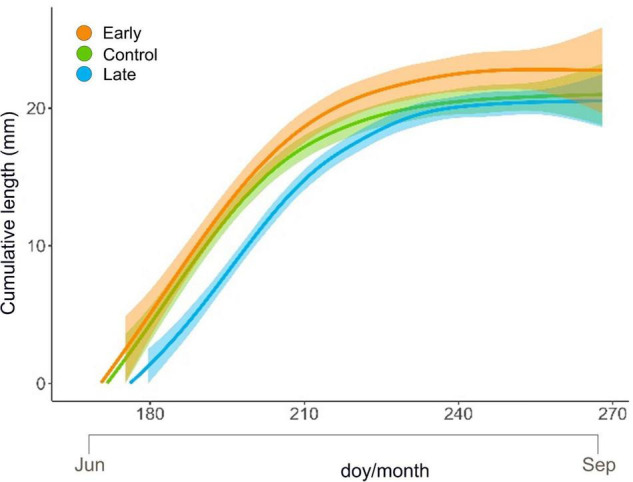
GAM modelled elongation trend (mm) with 95% confidence intervals of *Juniperus communis* new shoots over the growing season for the control (green), early-melt (orange) and late-melt (blue) treatment.

Shoot primary growth followed a sigmoidal trend for all treatments and all years (Figure 3 and Supplementary Figure 2). At the end of the growing season, the final length was 22.80 ± 3.23 mm (mean ± SE) for E-shrubs, 21.02 ± 2.33 mm for C-shrubs and 20.55 ± 2.07 for L-shrubs (Figure 3 and Supplementary Table 2).

All the predictive variables considered in the model had a significant effect on shoot elongation (Table 1).

**TABLE 1:** Outcome of GAM model applied to elongation as an independent variable in relation to year, shrub, stem, and snow treatment.

	DF	*F*-value	*p*-value
Treatment	2	4.12	<2e-16
Year	2	64.07	0.02
Shrub	4	8.76	4.89e-07
Shrub:Stem	31	13.71	<2e-16
Shrub:Stem:Branch	66	9.932	<2e-16
s (doy):E	5.02	156.4	<2e-16
s (doy):C	4.89	115.9	<2e-16
s (doy):L	4.73	147.2	<2e-16

*Degrees of freedom (DF), Fisher value (F-value), and p-value are shown.*

Maximum growth rate, computed as the 1st derivate of GAM-modelled elongation curves, was 0.55, 0.54, and 0.50, and it occurred on day 183, 182, and 196, for E-, C- and L-plants, respectively (Supplementary Table 3).

The beginning of shoot elongation did not reflect the number of days of snow removal between treatments. In fact, considering the three years together, despite the marked difference in the dates of snow disappearing between treatments (Figure 2 and Supplementary Table 1), E- and C-shoots started to grow in June with just a mean difference across the 3 years of 1 day (doy 170 for the E and 171 for the C) and C and L with a difference of 5 days (doy 176 for the L).

Comparing the elongation dynamic between the three years, while the control remained stable both in terms of maximum elongation rate and final length, the E- and L-plants did not feature any clear trend. However, for the manipulated groups, we observed a progressive reduction of the inter-individual and inter-group variability from the 1st to the 3rd year (Supplementary Figure 2 and Table 2).

**TABLE 2:** *F*-value, *p*-values of the linear mixed-effects models (nested ANOVA), and degrees of freedom (DF) of stomatal density, leaf area, leaf dry weight, leaf soluble sugars and leaf starch content in 2018, 2019, 2020, and averaging these 3 years (mean).

	Year	DF	*F*-value	*p*-value ANOVA
Stomatal density	2018	2–68	1.26	0.29
	2019	2–68	1.25	0.29
	2020	2–60	2.54	0.09
	mean	2–210	4.07	0.02
Leaf area	2018	2–427	6.89	0.001
	2019	2–447	7.97	<0.001
	2020	2–450	4.74	0.01
	mean	2–1338	18.66	<0.001
Leaf dry weight	2018	2–7	1.50	0.29
	2019	2–8	0.21	0.81
	2020	2–8	0.28	0.76
	mean	2–37	1.94	0.16
Soluble NSC	jun_2018	2–8	1.54	0.27
	jun_2019	2–8	2.07	0.19
	jun_2020	2–7	10.52	0.01
	mean_jun	2–37	2.80	0.07
	sep_2018	2–7	1.84	0.23
	sep_2019	2–7	21.04	0.001
	sep_2020	2–6	14.78	0.005
	mean_sep	2–34	1.60	0.22
Starch	jun_2018	2–7	29.73	0.004
	jun_2019	2–8	54.84	<0.001
	jun_2020	2–8	33.05	<0.001
	mean_jun	2–37	92.82	<0.001
	sep_2018	2–8	1.47	0.29
	sep_2019	2–8	2.20	0.17
	sep_2020	2–7	12.50	0.00
	mean_sep	2–37	3.17	0.05

*Non-structural carbohydrate (NSC) results refer to June (Jun) and September (Sep). Equal means are tested between early-snowmelt, control, and late-snowmelt treatments.*

### Treatment Effects on Leaf Traits

**FIGURE 4:**
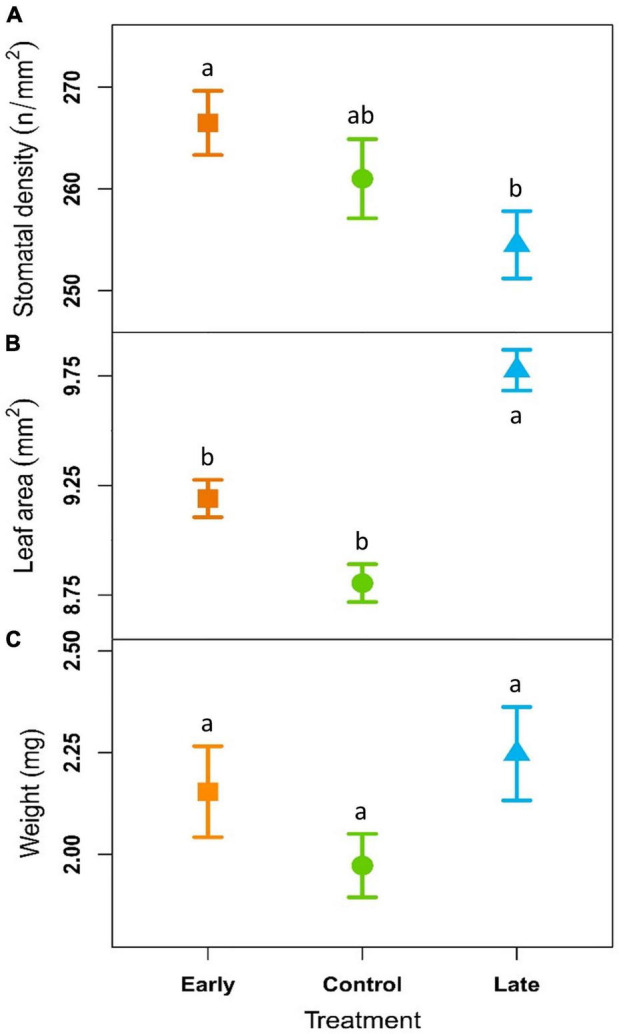
Outcome of linear mixed-effects models (nested ANOVA) of leaf traits. Mean ± SE stomatal density (**A**; n°/mm^2^), needle area (**B**; mm^2^) and dry weight per leaf (**C**; mg) of early-melt (orange), control (green), and late-melt (blue) shrubs in average from 2018 to 2020. Different letters indicate significant differences between groups (*p* < 0.05).

Stomatal density was 266 ± 4, 260 ± 4, and 254 ± 4 n°/mm^2^ for the E-, C- and L-groups, respectively. These values were not significantly different considering the single years and remained almost constant across the years for all the treatments. However, considering the whole period (2018–2020) stomatal density was significantly higher in E-plants compared to L-plants (Figure 4A, Table 2, and Supplementary Table 4).

Leaf area was on average 9.19 ± 0.09, 8.80 ± 0.09, and 9.78 ± 0.09 mm^2^ for the E-, C-, and L groups (Figure 4B and Supplementary Table 4) respectively, with significant difference between treatments considering both single years and all the three years together (Table 2). L-group had a larger leaf area than C for all time periods and a larger leaf area than E-plants in 2019 and the period 2018–2020. In addition, the leaf area did not present any trend over time but had a tendency to reduce the differences between treatments across the years and showed a step-like increase for all treatments from 2019.

Needle dry weight resulted 2.15 ± 0.11 mg for E-groups, 1.97 ± 0.08 mg for C and 2.25 ± 0.11 mg for the L (Figure 4C and Supplementary Table 4). This trait highlights no significant differences between treatments and no trend over time (Table 2).

Regarding SLA, no significant differences emerged between treatments (*p*-value = 0.82; Supplementary Figure 4).

### Treatment Effects on Non-structural Carbohydrates

**FIGURE 5:**
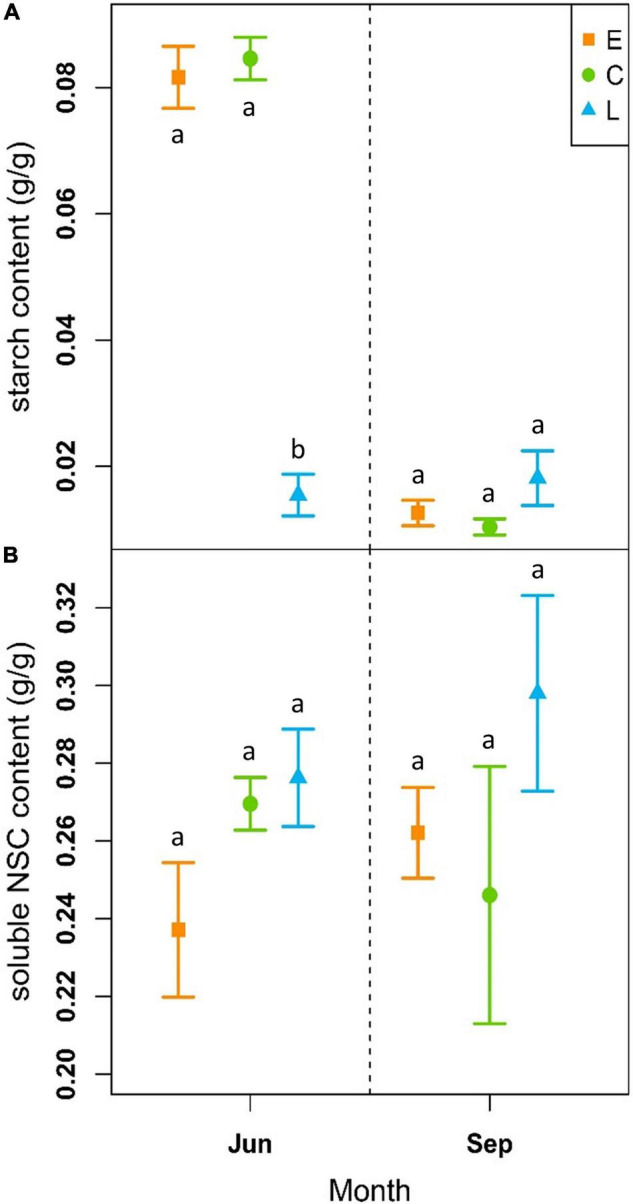
Outcome of linear mixed-effects models (nested ANOVA) of NSC leaf content. Mean ± SE starch (g/g; **A**) and NSC content (g/g; **B**) of early-melt (E; orange), control (C; green), and late-melt (L; blue) shrubs, considering the mean from 2018 to 2020, measured in June and September. Different letters indicate significant differences between groups (*p* < 0.05).

At the beginning of the growing season (June), soluble NSC content was on average 0.24 ± 0.02 g/g for E, 0.27 ± 0.01 g/g for C and 0.28 ± 0.01 g/g for the L-plants, while at the end of the vegetative period (September), it was 0.26 ± 0.01 g/g, 0.25 ± 0.03 g/g and 0.30 ± 0.02 g/g for the E, C and L, respectively (Figure 5B and Supplementary Table 6). The models revealed no significant differences between treatments neither at the beginning nor at the end of the growing season when considering the period 2018–2020 (Table 2). However, in June sugar content was slightly lower in E-shrubs in contrast to other treatments, even if not significantly (Figure 5B). In 2020, E-shrubs showed significantly lower soluble NSC content than the other two groups at the beginning of the season. Moreover, at the end of the growing season, C-plants and E-plants showed lower sugar content in 2019 and 2020, respectively (Table 2 and Supplementary Figure 5A).

On the contrary, starch content showed significant differences between treatments at the beginning of the season, but not at the end, for both single years and the whole period. The only exception is at the end of the 2020 season when L-plants have higher starch content than the other two groups. At the beginning of the season, L-plants had significantly less starch (Table 2 and Supplementary Figure 5A). Starch content at the beginning of the vegetative season was 0.08 ± 0.005 g/g for E, 0.08 ± 0.003 g/g for C and 0.02 ± 0.03 g/g for L, while at the end it was 0.01 ± 0.002 g/g for E, 0.01 ± 0.01 g/g for C and 0.02 ± 0.004 g/g for L (Figure 5A and Supplementary Table 5).

Non-structural carbohydrates between treatments did not show a clear trend over time or a specific response to treatments over years. However, variability of starch content increased within groups and decreased between treatments (Supplementary Figure 5 and Supplementary Tables 5, 6).

## Discussion

With a manipulative 3-year experiment, we monitored the immediate and carryover effects of different snowpack duration on primary growth, leaf traits and NSC leaf content on *J. communis* growing above the treeline.

According to our initial hypothesis, a few traits were affected by snow cover duration already from the first year, namely primary growth, leaf area and leaf starch content. However, in contrast to our expectations, the differences between treatments for these traits seemed to decrease progressively, except for soluble NSC content. In fact, soluble NSC, together with stomatal density showed an overall response to different snow cover treatments only at the end of the experiment or looking at the whole period. Instead, other traits (growth rate, leaf dry weight and SLA) seemed rather unaffected by the varying persistence of the snow cover.

Above the alpine treeline, early frost is generally considered a crucial stressor, especially on early-exposure sites (Wheeler et al., 2014). This suggests that earlier exposure of plants to air compared to natural snowpack melting might increase the risk of damages due to freezing events, yet with the concurrent effect of accelerating the onset of the growing season (Björk and Molau, 2007). At this elevation, freezing temperatures can occur during the vegetative season, highlighting the lack of a frost-free period (Larcher and Wagner, 2009; Neuner, 2014). Common juniper living in harsh conditions is well-adapted to frost (Thomas et al., 2007), and freezing risk might not be considered as a crucial limiting factor for E-plants in this experiment. Additionally, juniper needles withstood temperatures of up to −10°C (Taschler and Neuner, 2004) also during the summer, when frost resistance is usually lower compared to winter or early spring (Neuner, 2014). Accordingly, we did not observe any significant sign of frost damage, like dead branches or needles, during the three years. The absence of frost damages on the morphology of the needles, besides the shrub resistance to frost, could also be related to the relatively low number of random events experienced by plants during the experiment, such as intense frost (Supplementary Figure 1A).

The low frost impact on E-plants was also confirmed by the fact that these started to elongate earlier, at a higher rate throughout the summer to finally reach longer extension, 8.5% more than control shoots, at the end of the season (Figure 3 and Supplementary Table 2). This may suggest that early snow-cover removal has a potential direct effect on the length of the vegetation period with E-plants benefiting from earlier exposure to daylight. Since stomata are the regulator of photosynthetic performance (Hetherington and Woodward, 2003) and higher stomatal density might improve plant productivity (Schlueter et al., 2003), we may also speculate that the observed enhanced elongation could result from the higher stomatal density detected on E-individuals. In fact, the stomatal density of developing leaves, besides CO_2_ concentration and plant hormones, is positively related to temperature (Schlueter et al., 2003) and irradiance received by mature leaves (P. W. Hetherington and Woodward, 2003; Thomas et al., 2004; Casson and Gray, 2008). E- and L-plants showed a slight but divergent response in terms of stomatal density when considering the whole period (Figure 4A). Accordingly, considering that light is one of the key varying factors between treatments, we may hypothesise that E-plants received overall more light than L-plants, letting more epidermal cells develop into stomata (Schoch et al., 1980), and vice versa.

Late melting-shoots, despite the delayed and lower peak of elongation rate, reached maximal length just 2.2% shorter than controls at the end of the vegetative period. The reduced growth is in line with the detrimental effect of snow cover persistence on long-term radial growth, that similarly to primary growth is linked to carbon gain, of common juniper across the Alps (Carrer et al., 2019). However, *J. communis* showed high plasticity in growth phenology that could potentially allow it to tune its growth rates during the growing seasons to better cope with the peculiar pace of climate variability, and modulate carbon investment accordingly (Tumajer et al., 2021). Plasticity in the growth rate could be the key attribute permitting L-plants to recover the growth lag in respect to control and E-plants. This growth acclimation is shared with other shrub species able to colonize a wide range of environments, such as *Salix artica* and *Rhododendron ferrugineum* growing in heat-limited environments, of which radial growth is negatively affected by snow persistence (Schmidt et al., 2010; Francon et al., 2020). On the contrary, early snowmelt did not boost the primary growth of other alpine species, such as *Salix herbacea, Vaccinium myrtillus, Vaccinium uliginosum*, and *Loiseleuria procumbens* (Wheeler et al., 2014). These differences could likely derive from different species and growing conditions, besides different experimental methodologies that can highlight or attenuate such a result. In general, the responses in terms of primary growth of shrubs to changing snow persistence seem rather heterogeneous (Wipf and Rixen, 2010), highlighting the need for species-specific studies and stressing the role of microclimatic conditions in promoting growth (Myers-Smith et al., 2015; Tumajer et al., 2021).

Nevertheless, the upswing of L-shoot growth may also be supported by larger leaves, that is larger photosynthetic area found in L-plants, as they increase the radiation absorption and therefore carbon input (Chapin et al., 2002). In fact, L-shrubs produced significantly larger leaves than both C- and E-shrubs (Figure 4B and Table 2), featuring a plastic response in this leaf trait in relation to altered snowmelt timings, in accordance with our hypothesis. The reason behind larger leaves could be connected to the mean warmer conditions experienced by L-shrubs that should favour larger leaf area. Despite the shorter growing season, L-shrubs emerged from the snowpack in correspondence with the peak mean air temperature (Figure 2 and Supplementary Figure 1A). Similar investigations on *S. herbacea* in the Alps (Sedlacek et al., 2015) and, more in general, across the tundra biome found a similar outcome, with a positive association between temperature and leaf area (Bjorkman et al., 2018). Likely, together with the larger photosynthetic area, also higher efficiency of light may have enhanced L-plants growth, in line with the response of Arctic tundra vegetation to shorter growing season caused by prolonged snow cover (Bosiö et al., 2014). In parallel, the leaf dry weight of L-plants was just slightly higher but not significantly affected by snow removal (Figure 4C and Table 2). This result, together with the larger L-needles, seems in accordance with the tendency of leaves to reduce their construction costs (represented by dry weight) and increase the photosynthetic rate with increasing latitude to maximally exploit the short growing season (Tian et al., 2016).

In general, NSC content in C-plants is consistent with seasonal data proposed by Rabska et al. (2020), even though, surprisingly, another study on the same species proposed a soluble NSC content 100-fold lower (Magaña Ugarte et al., 2021). The high level of soluble NSC we measured can be attributed to the time of collection that corresponds to the maximal diurnal photosynthetic rate (Bickford et al., 2009). Nonetheless, we consider that the observed differences in NSC should be attributed to the plant response to the distinct snowpack duration. In fact, the evergreen common juniper does not rely on new needles to perform photosynthesis when it is out from snow cover and its photosynthetic capacity does not dramatically vary seasonally (Rabska et al., 2021).

Plants exposed to abiotic stresses often accumulate starch that can be remobilized for energy and carbon supply, under conditions of photosynthesis limitation (Thalmann and Santelia, 2017). However, starch in leaves is a transitory form accumulated in the chloroplast during active photosynthesis. Focusing on the lower level of starch, observed in leaves of L-plants in late spring (Figure 5), we may notice that these plants likely developed a good level of photosynthetic rate; in fact, no significant differences were observed in the sugar pool. This suggests that transitory starch was poorly accumulated in the chloroplast because sucrose is promptly exported to the sink organ actively growing, e.g., buds (Ray and Savage, 2021). Consistently, L-plants showed a high growth rate in late spring, i.e., when NSCs were measured (see Figure 3 doy 200–210).

Moreover, in our case, the primary growth dynamic seemed significantly influenced not just by the treatment but also by other factors related to time (year) and the individual (Table 1). For example, selected stem and branch, besides shrub, highlighted a significant effect on primary growth (Table 1), suggesting that the intra-individual variability could be potentially as high, or even higher, than the intraspecific variability. This may reflect the intraspecific homogeneity, at least for some physiological traits, such as functional traits related to hydraulic efficiency, of this *taxon* (Unterholzner et al., 2020).

We found a remarkable converging tendency between treatments over years for primary growth dynamics, leaf area, leaf dry weight and, partially, also for starch leaf content. This may suggest a plant’s progressive acclimation to the new conditions across time. On one hand, L-plants seem to enhance their efficiency in exploiting the shorter growing seasons, on the other hand, E-plants seem to lose their advantage of experiencing longer growing seasons. In the long term, E-plants may be affected by the harsher soil conditions, in terms of microbial communities, when exposed to early snowmelt (Broadbent et al., 2021), and in parallel soil under prolonged snow cover may increase N soil availability (Semenchuk et al., 2015).

These results are even more remarkable when considering that, despite the difference of snow-removal days between treatments (Figure 3), shrubs started to grow within just a few days of each other (E/L shoots 1/5 days before/after the controls), when daily mean air temperature was around 10°C (Figure 2A). Processes needed for alpine plants to grow to require a certain temperature threshold, below which they do not activate (Körner, 2003). Since snowpack duration, in parallel with temperature increase, is predicted to drastically shrink in the next decades in both Alpine (Steger et al., 2013) and Arctic regions (Meredith et al., 2019), a substantial phytomass increase may be expected. This is consistent with the overall, even if heterogeneous, current tundra shrub encroachment and with the ca. 20% biomass increase detected in the Northern hemisphere in the recent decades (Epstein et al., 2012; Frost et al., 2020). Nonetheless, future predictions are far from being straightforward considering the high plasticity of this species and the potential parallel change of other external factors as a consequence of snow regime change. Finally, since changes in growing season length may also influence the interplay between plants and pathogens, pollinators or herbivores, we could expect potential reshaping in trophic interactions (Roy et al., 2004; Høye et al., 2013; Sedlacek et al., 2015). Also, even if individual performance may benefit from the future climate, predicting potential vegetation shifts would benefit from also considering climatic responses of shrub community. For instance, the benefit of warmer temperature for recruitment of Juniper species at high elevation may be hindered by water stress, highlighting the role of abiotic interaction on vegetation dynamics (Sigdel et al., 2021). Indeed, common juniper (but also other plants) growing on snow beds could benefit from the additional soil water available due to, e.g., late snowmelt. Still, additional studies are needed to disentangle the role of the different abiotic (environmental and climatic) and biotic (e.g., competition) components to get insight into shrubs’ responses. Therefore, we encourage future studies to focus not only on the individual but also on the community and ecosystem levels.

## Data Availability Statement

The raw data supporting the conclusions of this article will be made available by the authors on request.

## Author Contributions

MC conceived the experiment and designed the study with input from the co-authors. LU, ALP, RD, RM, and MC contributed to the experiment maintenance, collected samples, and performed fieldwork activities. LU measured stomatal density and leaf area, analysed the data with input from ALP and FB, and designed tables and figures. VC, SG, and FB contributed to run non-structuralcarbohydrates measurements. LU interpreted the results with inputs from MC, ALP, VC, SG, and FB and drafted the 1st version of the manuscript with inputs from MC and the contribution of all other co-authors. All authors contributed to the article and read and approved the submitted version.

## Conflict of Interest

The authors declare that the research was conducted in the absence of any commercial or financial relationships that could be construed as a potential conflict of interest.

## Publisher’s Note

All claims expressed in this article are solely those of the authors and do not necessarily represent those of their affiliated organizations, or those of the publisher, the editors and the reviewers. Any product that may be evaluated in this article, or claim that may be made by its manufacturer, is not guaranteed or endorsed by the publisher.
