# Supplementary material for: Transient Effects of Snow Cover Duration on Primary Growth and Leaf Traits in a Tundra Shrub
Source: Front Plant Sci. 2022 Apr 5;13:822901. doi: 10.3389/fpls.2022.822901 (PMC9037292; doi:10.3389/fpls.2022.822901)
Supplement: Supplementary file 1 [file Data_Sheet_1.docx]

Supplementary material

Transient effects of snow cover duration on primary growth and leaf traits in a tundra shrub

Lucrezia Unterholzner^1^*, Angela Luisa Prendin^1,2^, Raffaella Dibona^1^, Roberto Menardi^1^, Valentino Casolo^3^, Sara Gargiulo^3,4^, Francesco Boscutti^3^, Marco Carrer^1^

^1^Department of Land Environment Agriculture and Forestry, University of Padova, Legnaro, Italy;

^2^ Department of Biology, Ecoinformatics and Biodiversity, Aarhus University, Aarhus C, Denmark

^3^ Department of Agricultural Food Environmental Animal Sciences, University of Udine, Udine, Italy;

^4^ Department of Life Sciences, University of Trieste, Trieste, Italy;

* Correspondence:

Lucrezia Unterholzner

lucrezia.unterholzner@phd.unipd.it


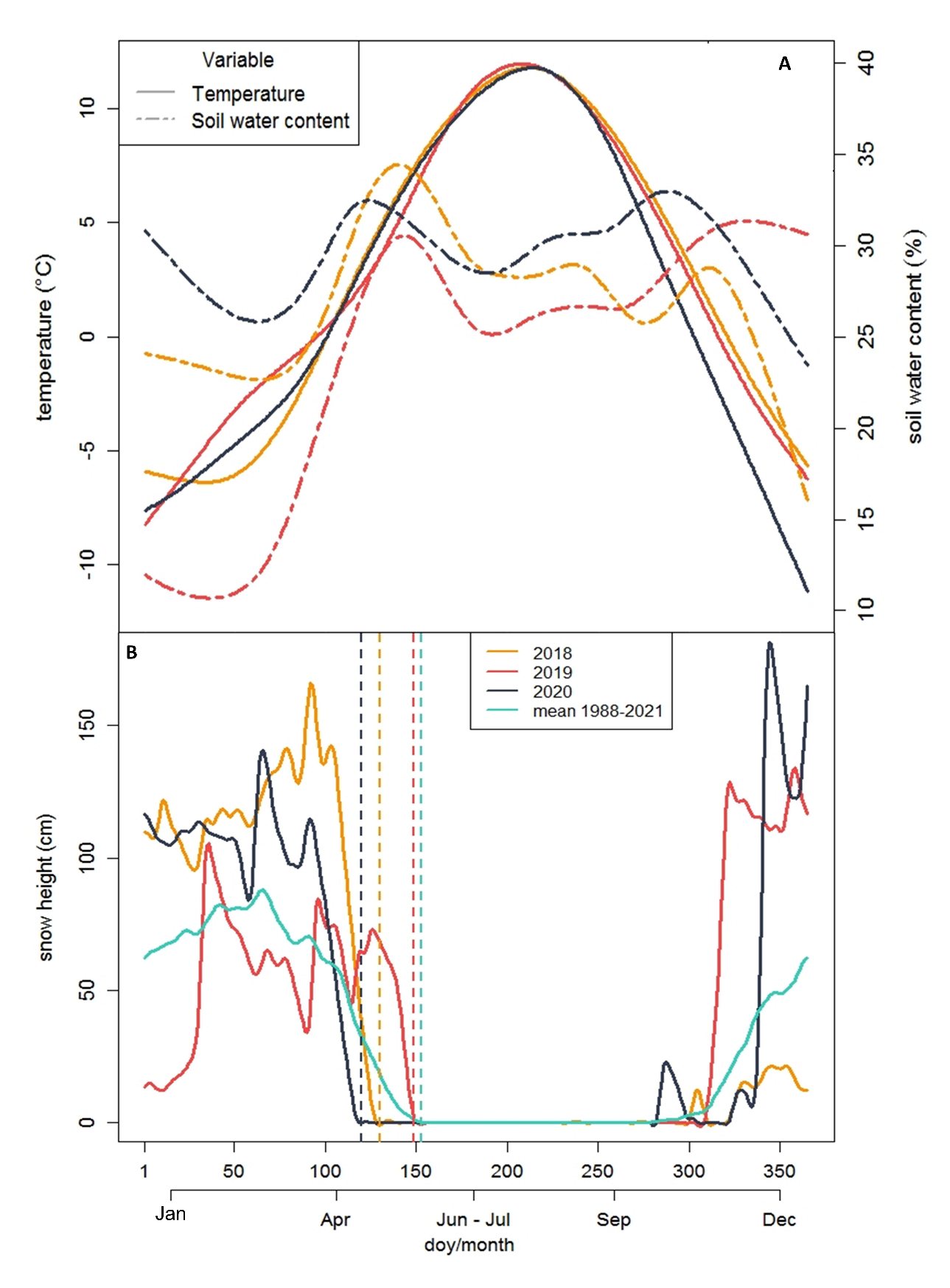


Supplementary Figure 1: Mean air temperature (°C), soil water content (%) over the days of the year (doy) and over months considering the period 2018-2020, registered at Giau Pass (A) and snow height (cm) recorded at the Falzarego Pass weather station in the period 2018-2020 and in average for the 1988-2020 period (B). Data are interpolated through spline function. Dashed lines represent the doy on which snow was completely melt.


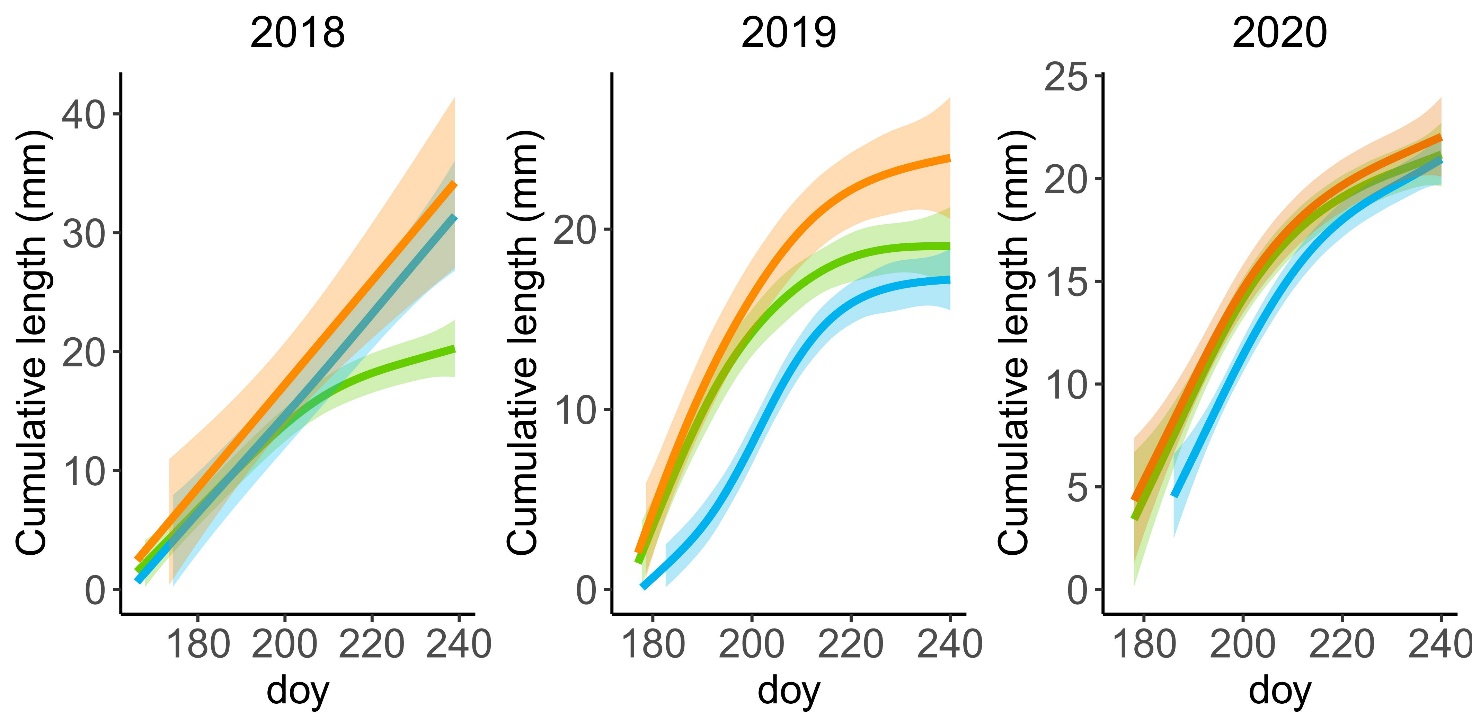


Supplementary Figure 2: Elongation trend of 2018, 2019, 2020 of Juniperus communis L. shoots over day of the year (doy) for the control (green), uncovered (orange) treatment and covered treatment (blue). Solid line is the elongation trend modelled by GAM model, coloured band around solid line represents the confidence interval at 95% of probability.


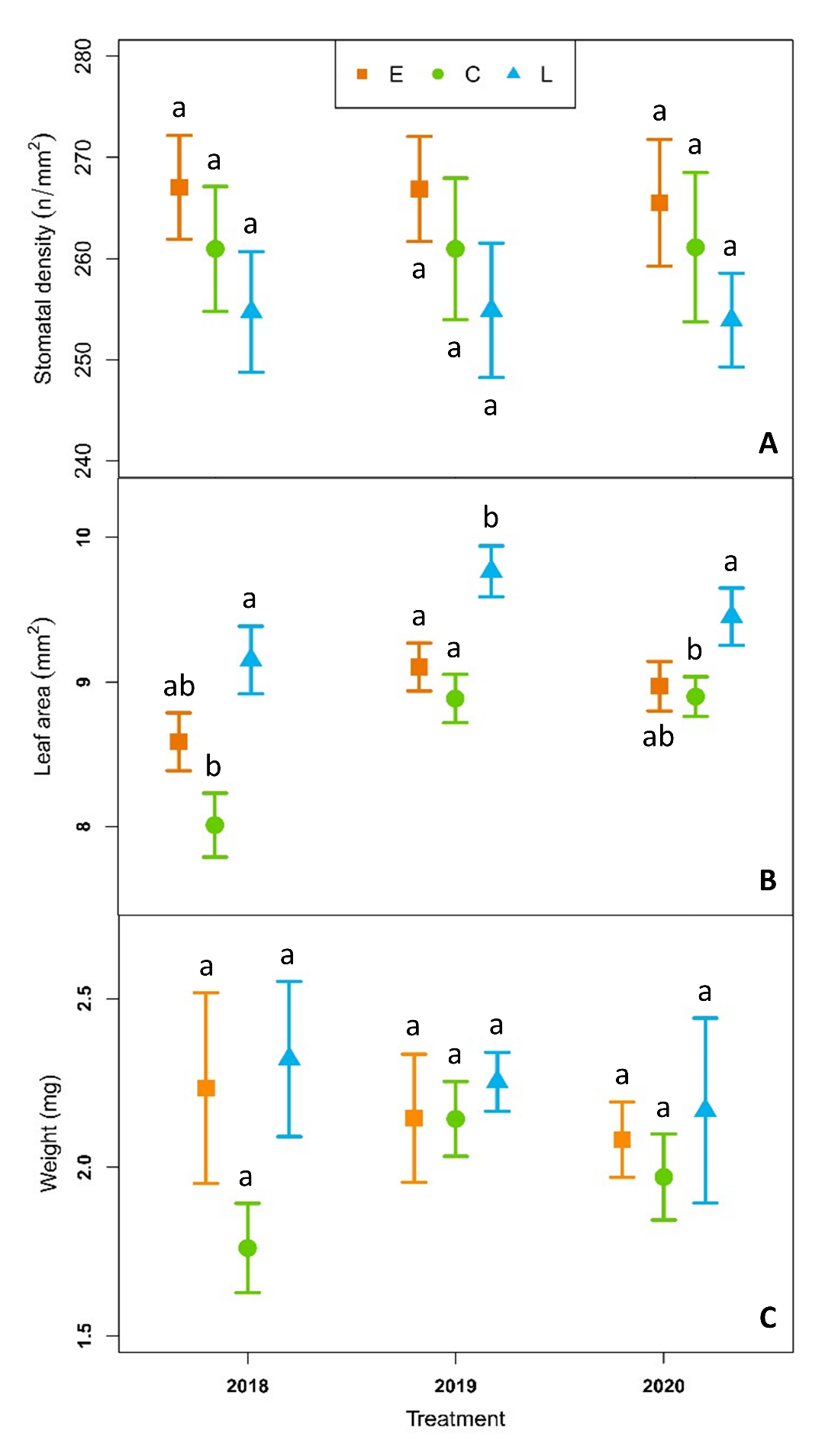


Supplementary Figure 3: Outcome of linear mixed-effects models (nested ANOVA) of leaf traits. Mean ± SE stomatal density (A; n°/mm2), needle area (B; mm2) and dry weight per leaf (C; mg) of early-melt (orange), control (green) and late-melt (blue) shrubs in 2018, 2019 and 2020. Equal letters indicate that there are no significant differences between groups (p>0.05).


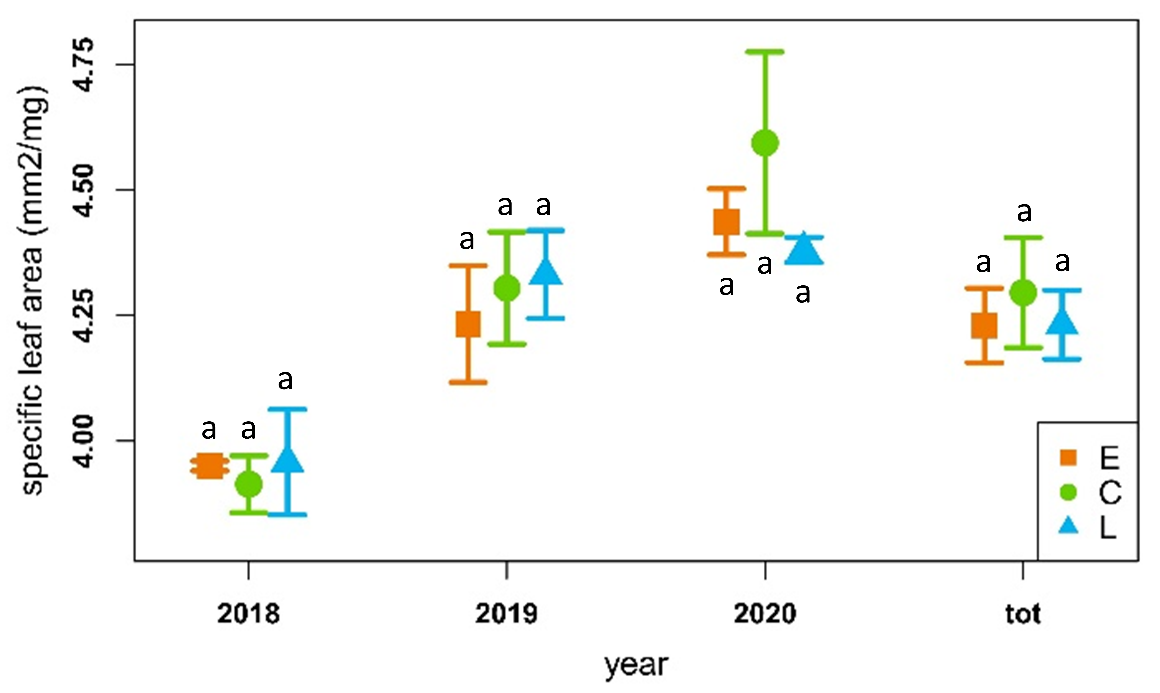


Supplementary Figure 4: Outcome of linear mixed-effects models (nested ANOVA) of specific leaf area. Mean ± SE specific leaf area (mm^2^/mg) of uncovered (orange), control (green) and covered (blue) shrubs, in 2018, 2019, 2020 and in average from 2018 to 2020 (mean).


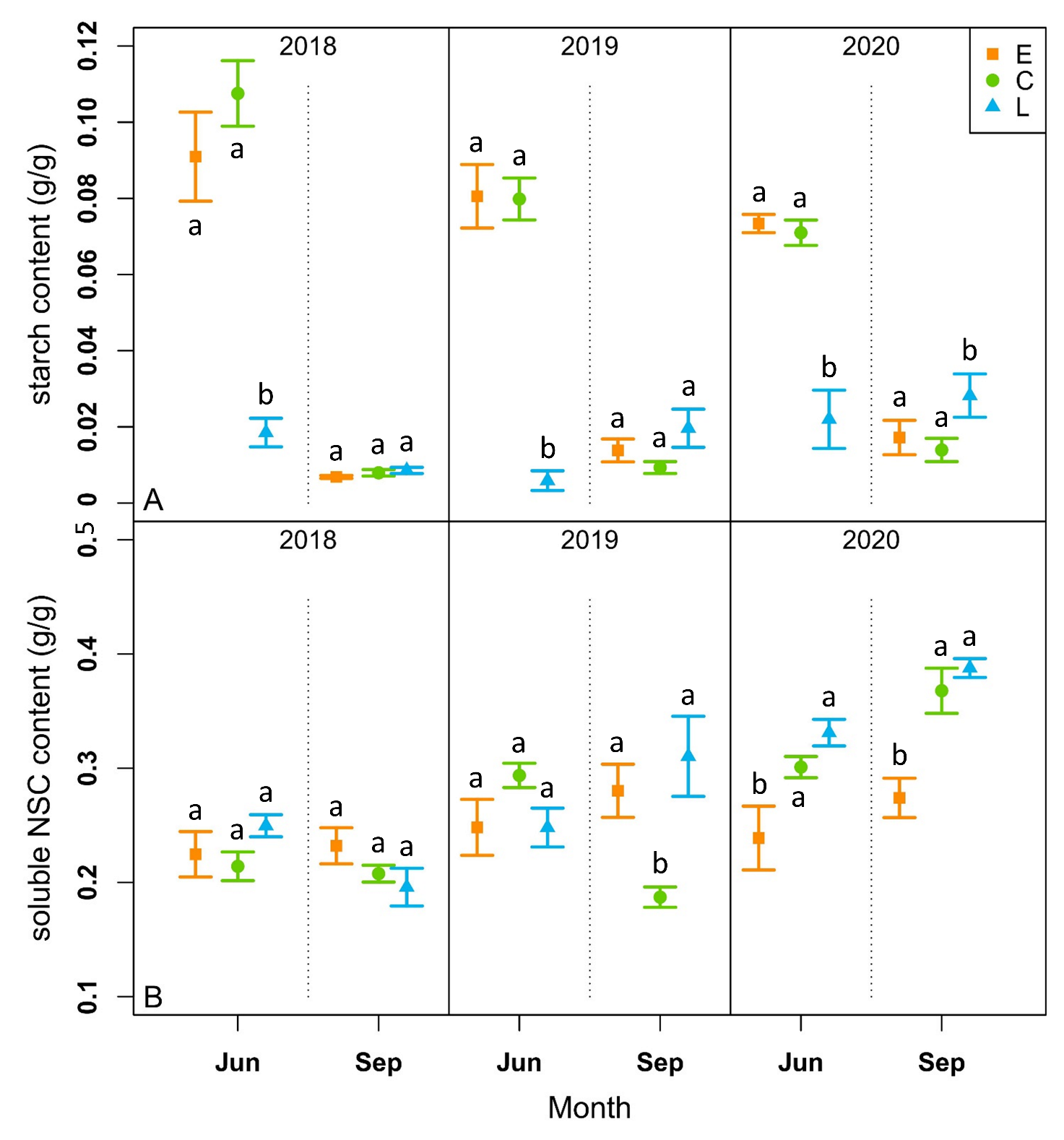


Supplementary Figure 5: Outcome of linear mixed-effects models (nested ANOVA) of leaf NSC content. Mean ± SE starch (g/g) and soluble sugars (g/g) leaf content of early-melt (E; orange), control (C; green) and late-melt (L; blue) shrubs, in 2018, 2019, 2020 measured at the beginning (June) and at the end (September) of the growing season.

| year | E-C | L-C | E-L |
| --- | --- | --- | --- |
| 2018 | 53 | 35 | 88 |
| 2019 | 95 | 37 | 132 |
| 2020 | 39 | 27 | 66 |
| mean | 62 | 33 | 95 |

Supplementary Table 1: Difference of days of snow removal for early snowmelt (E), control (C) and late snowmelt (L) treatments in 2018, 2019, 2020 and in average for these three years (mean).

| Max length (mm) | SE (mm) | treatment | year |
| --- | --- | --- | --- |
| 20.31 | 2.38 | C | 2018 |
| 31.86 | 2.96 | L |  |
| 32.77 | 6.71 | E |  |
| 19.21 | 2.41 | C | 2019 |
| 17.43 | 2.40 | L |  |
| 24.29 | 4.48 | E |  |
| 21.47 | 1.82 | C | 2020 |
| 21.38 | 1.49 | L |  |
| 22.48 | 2.39 | E |  |
| 21.02 | 2.33 | C | mean |
| 20.55 | 2.07 | L |  |
| 22.80 | 3.25 | E |  |

Supplementary Table 2: Maximum length (mm) reached and SE of primary shoots of Juniperus communis growing at Giau pass, measured in 2018, 2019, 2020 ad in average for these three years (mean), under early snowmelt (E), control (C) and late snowmelt (L) treatments.

| Max slope | treatment | year | doy |
| --- | --- | --- | --- |
| 0.38 | C | 2018 | 179 |
| 0.44 | L |  | 223 |
| 0.45 | E |  | 185 |
| 0.65 | C | 2019 | 177 |
| 0.51 | L |  | 201 |
| 0.70 | E |  | 177 |
| 0.50 | C | 2020 | 184 |
| 0.49 | L |  | 193 |
| 0.47 | E |  | 185 |
| 0.54 | C | mean | 182 |
| 0.50 | L |  | 196 |
| 0.55 | E |  | 183 |

Supplementary Table 3: Maximal slope of the elongation curve modelled by GAM model of Juniperus communis growing at Giau pass, measured in 2018, 2019, 2020 ad in average for these three years (mean), under early snowmelt (E), control (C) and late snowmelt (L) treatments.

|  | year | treatment | mean | SE | n |
| --- | --- | --- | --- | --- | --- |
| STOMATAL DENSITY | 2018 | E | 268 | 6 | 25 |
|  |  | C | 260 | 6 | 25 |
|  |  | L | 254 | 6 | 25 |
|  | 2019 | E | 266 | 6 | 25 |
|  |  | C | 260 | 6 | 25 |
|  |  | L | 254 | 6 | 25 |
|  | 2020 | E | 266 | 6 | 24 |
|  |  | C | 262 | 8 | 18 |
|  |  | L | 254 | 4 | 25 |
|  | tot | E | 260 | 2 | 114 |
|  |  | C | 254 | 4 | 110 |
|  |  | L | 254 | 2 | 120 |
| LEAF AREA | 2018 | E | 8.59 | 0.2 | 154 |
|  |  | C | 8.01 | 0.22 | 151 |
|  |  | L | 9.15 | 0.23 | 129 |
|  | 2019 | E | 9.1 | 0.17 | 168 |
|  |  | C | 8.89 | 0.17 | 143 |
|  |  | L | 9.77 | 0.18 | 143 |
|  | 2020 | E | 8.97 | 0.17 | 159 |
|  |  | C | 8.9 | 0.14 | 157 |
|  |  | L | 9.45 | 0.2 | 141 |
|  | tot | E | 9.19 | 0.085 | 682 |
|  |  | C | 8.8 | 0.086 | 661 |
|  |  | L | 9.78 | 0.093 | 635 |
| DRY WEIGHT | 2018 | E | 2.23 | 2.83 | 153 |
|  |  | C | 1.76 | 1.32 | 151 |
|  |  | L | 2.32 | 2.30 | 131 |
|  | 2019 | E | 2.15 | 1.90 | 169 |
|  |  | C | 2.14 | 1.10 | 141 |
|  |  | L | 2.25 | 8.73 | 143 |
|  | 2020 | E | 2.08 | 1.12 | 154 |
|  |  | C | 1.97 | 1.28 | 157 |
|  |  | L | 2.17 | 2.74 | 150 |
|  | mean | E | 2.15 | 1.12 | 476 |
|  |  | C | 1.97 | 7.79 | 449 |
|  |  | L | 2.25 | 1.15 | 424 |

Supplementary Table 4: Mean and SE of stomatal density(n°/mm2), area per leaf (mm2) and dry weight per leaf (mg) of Juniperus communis growing at Giau pass, measured in 2018, 2019, 2020 ad in average for these three years (mean), under early snowmelt (E), control (C) and late snowmelt (L) treatments. N is sample depth.

|  | year | month | treatment | mean | SE | n |
| --- | --- | --- | --- | --- | --- | --- |
| STARCH | 2018 | jun | E | 0.091 | 0.012 | 5 |
|  |  |  | C | 0.108 | 0.009 | 5 |
|  |  |  | L | 0.019 | 0.004 | 5 |
|  |  | set | E | 0.007 | 0.000 | 5 |
|  |  |  | C | 0.008 | 0.001 | 5 |
|  |  |  | L | 0.009 | 0.001 | 5 |
|  | 2019 | jun | E | 0.081 | 0.008 | 5 |
|  |  |  | C | 0.080 | 0.006 | 5 |
|  |  |  | L | 0.006 | 0.003 | 5 |
|  |  | set | E | 0.014 | 0.003 | 5 |
|  |  |  | C | 0.009 | 0.002 | 5 |
|  |  |  | L | 0.020 | 0.005 | 5 |
|  | 2020 | jun | E | 0.073 | 0.003 | 5 |
|  |  |  | C | 0.071 | 0.003 | 5 |
|  |  |  | L | 0.022 | 0.008 | 5 |
|  |  | set | E | 0.017 | 0.005 | 5 |
|  |  |  | C | 0.014 | 0.003 | 5 |
|  |  |  | L | 0.028 | 0.006 | 5 |
|  | mean | jun | E | 0.08 | 0.005 | 15 |
|  |  |  | C | 0.08 | 0.003 | 15 |
|  |  |  | L | 0.02 | 0.003 | 15 |
|  |  | set | E | 0.01 | 0.002 | 15 |
|  |  |  | C | 0.01 | 0.001 | 15 |
|  |  |  | L | 0.02 | 0.004 | 15 |

Supplementary Table 5: Mean and SE of starch leaf content (g/g) of Juniperus communis growing at Giau pass, measured in 2018, 2019, 2020 ad in average for these three years (mean in June and September), under early snowmelt (E), control (C) and late snowmelt (L) treatments. n is sample depth.

|  | year | month | treatment | mean | SE | n |
| --- | --- | --- | --- | --- | --- | --- |
| SOLUBLE SUGARS | 2018 | jun | E | 0.225 | 0.020 | 5 |
|  |  |  | C | 0.214 | 0.013 | 5 |
|  |  |  | L | 0.250 | 0.010 | 5 |
|  |  | set | E | 0.232 | 0.016 | 5 |
|  |  |  | C | 0.208 | 0.007 | 5 |
|  |  |  | L | 0.196 | 0.017 | 5 |
|  | 2019 | jun | E | 0.248 | 0.025 | 5 |
|  |  |  | C | 0.294 | 0.011 | 5 |
|  |  |  | L | 0.248 | 0.017 | 5 |
|  |  | set | E | 0.280 | 0.023 | 5 |
|  |  |  | C | 0.187 | 0.009 | 5 |
|  |  |  | L | 0.310 | 0.035 | 5 |
|  | 2020 | jun | E | 0.239 | 0.028 | 5 |
|  |  |  | C | 0.301 | 0.009 | 5 |
|  |  |  | L | 0.331 | 0.012 | 5 |
|  |  | set | E | 0.274 | 0.017 | 5 |
|  |  |  | C | 0.368 | 0.020 | 5 |
|  |  |  | L | 0.388 | 0.011 | 5 |
|  | mean | jun | E | 0.24 | 0.017 | 15 |
|  |  |  | C | 0.27 | 0.012 | 15 |
|  |  |  | L | 0.28 | 0.013 | 15 |
|  |  | set | E | 0.26 | 0.012 | 15 |
|  |  |  | C | 0.25 | 0.033 | 15 |
|  |  |  | L | 0.30 | 0.025 | 15 |

Supplementary Table 6: Mean and SE of soluble sugar leaf content (g/g) of Juniperus communis growing at Giau pass, measured in 2018, 2019, 2020 ad in average for these three years (mean in June and September), under early snowmelt (E), control (C) and late snowmelt (L) treatments. n is sample depth.
